# Diffuse connective tissue disorders in HIV-infected patients

**DOI:** 10.31138/mjr.29.3.148

**Published:** 2018-09-27

**Authors:** Anna Christoforidou, Nikolaos Galanopoulos

**Affiliations:** 1 Department of Haematology, University General Hospital of Alexandroupolis, Thrace, Greece; 2 Outpatient Department of Rheumatology, University General Hospital of Alexandroupolis, Thrace, Greece

**Keywords:** HIV, AIDS, rheumatic, Highly Active Anti-retroviral Therapy, HAART, Diffuse Infiltrative Lymphocytosis Syndrome, DILS, Lupus Erythematosus, Sjogren’s syndrome, Antiphospholipid Syndrome, vasculitis, connective tissue disorders, Behçet’s disease, Henoch-Schönlein purpura, cryoglobulins, Cryoglobulinemic Vasculitis, sicca, immune reconstitution inflammatory syndrome, immunosuppressant drugs

## Abstract

**Background:**

Human immunodeficiency virus (HIV) infection has been associated with various autoimmune disorders.

**Aim:**

To review the spectrum of diffuse connective tissue disorders (dCTD) in HIV-infected patients, in the context of highly active anti-retroviral therapy.

**Methods:**

Electronic search of the literature was performed using the terms HIV, AIDS, autoimmune, rheumatic/rheumatological, immune reconstitution inflammatory syndrome, Systemic Lupus Erythematosus, Diffuse Infiltrative Lymphocytosis Syndrome, Sjogren’s syndrome, vasculitis, Behçet’s disease, cryoglobulins, Henoch-Schönlein purpura, and antiphospholipid syndrome.

**Results:**

We reviewed the clinical manifestations, natural history and treatment of dCTDs, since the implementation of Highly Active Anti-Retroviral Therapy (HAART), and the emergence of new pathogenic mechanisms, such as the immune reconstitution inflammatory syndrome.

**Conclusions:**

Caution in differentiating clinical and laboratory findings of dCTDs from non-specific manifestations of acute and chronic HIV infection is warranted due to the common presentation. Patients with chronic infection and access to HAART have a normal life expectancy and dCTDs, although rare, must be correctly addressed. HAART alone or combined with immunosuppressive therapy result in favourable outcomes.

## INTRODUCTION

In patients with HIV (human immunodeficiency virus) infection, a variety of rheumatological manifestations have been reported. These complications range from myoskeletal disorders such as arthralgias/arthritis, myositis, osteonecrosis, osteoporosis, septic arthritis and osteomyelitis, to manifestations associated with connective tissue diseases.^1–5^ Before the widespread use of HAART (Highly Active Anti-Retroviral Therapy), up to 72% of patients manifested some kind of rheumatic complications,^5^ but in the recent literature, the incidence seems to be diminished and patterns have changed, with septic complications being the most prevalent,^6^ along with the immune reconstitution inflammatory syndrome (IRIS).^7–9^

In this review, we are focusing on diffuse connective tissue disorders (dCTD) only, with an emphasis on the post-HAART era. dCTDs may be diagnosed either preceding or in parallel to the HIV diagnosis, reflecting the immune system compromise. Various autoimmune diseases (AD) can also be a manifestation of IRIS, which occurs after HAART therapy, such as sarcoidosis, thyroid disease and vasculitis. In general, AD in HIV infected patients can develop by multiple mechanisms, including a direct role of the viral particles, immune complexes, molecular mimicry and the deregulation of B/T cell interaction. HAART can play a dual role: on one hand, reducing the autoimmune phenomena by restoring the immune regulation, and on the other hand, triggering the flare of a latent AD or the onset of a new one during the IRIS, mimicking a delayed hypersensitivity reaction to a foreign or self antigen. HIV infection can present with various symptoms and laboratory abnormalities that simulate dCTDs, so caution should be applied in interpreting a clinical presentation.

## METHODS

We performed an electronic literature review using the PubMed Journal database and Google Scholar. We searched for the terms: HIV, AIDS, autoimmune, rheumatic/rheumatological, immune reconstitution inflammatory syndrome, Systemic Lupus Erythematosus, Diffuse Infiltrative Lymphocytosis Syndrome, Sjogren’s syndrome, vasculitis, ANCA, antineutrophil cytoplasmic antibody (ANCA)-associated vasculitides, Behçet’s disease, cryoglobulins, Cryoglobulinemic Vasculitis, Henoch-Schönlein purpura, Antiphospholipid Syndrome, antiphospholipid antibodies, anticardiolipin antibodies. Review articles, clinical case studies, case series, epidemiological studies and original research articles, between the year 1985 and 02/28/2018, were selected.

## SYSTEMIC LUPUS ERYTHEMATOSUS

Systemic Lupus Erythematosus (SLE) is rare in the course of HIV patients.^1–3^ The real incidence is difficult to be estimated since there is an overlap of symptoms between SLE and HIV infection, and some cases of SLE are missed because manifestations are attributed to the viral infection and vice versa.

Both diseases can present with fever, myalgia, arthralgia or arthritis, skin rash, neurological manifestations, renal and other organ involvement. They may also have common laboratory abnormalities like anemia, leukopenia, lymphopenia, thrombocytopenia, autoantibodies, hypergammaglobulinemia and renal impairment.^10–13^ This association and the risk of misdiagnosis was already evident in the early period of HIV/AIDS recognition (Kopelman et al., 1988^14^) and provides an insight into the pathogenesis of both diseases. Although the current criteria for SLE diagnosis are highly specific, exclusion diagnosis should also be employed.

The observation that there are fewer HIV-infected patients with concomitant SLE than expected compared to the general population led to a more detailed examination of this occurrence. The mere demographic argument that HIV is more prevalent in males in contrast to SLE ceased to exist, since in sub-Saharan Africa and other parts of the world women comprise more than half of HIV-infected patients. According to Fox RA and Isenberg DA, the production of autoantibodies may be protective against HIV.^15^ There are multiple cases of patients with pre-existing SLE in whom the disease went into remission after advanced HIV infection.^15–18^ Some of these patients experienced a flare of the symptoms after the retroviral therapy, which might or not be associated with the increasing number of CD4+ lymphocytes and immune reconstitution.^18^ In patients developing SLE many years after HAART, such an association cannot be asserted.^19^

Iordache et al. found 52 patients with autoimmune diseases in a HIV patient population.^20^ Among those, 7 had been diagnosed with lupus. In 2 cases, SLE preceded HIV diagnosis; in 1 it was diagnosed simultaneously, and in the other 4, after viral elimination by HAART. Mean CD4 count at the time of SLE diagnosis was 369/mm,^3^ and most of them required immunosuppressive therapy. Modi et al. identified 13 patients from South Africa with concurrent SLE and HIV infection. In 6 of them, the diseases were diagnosed at the same time; 5 had pre-existing SLE, and in 2 of them, lupus developed at 30 and 35 months after HAART therapy.^18^ ANA titers ranged from 1:640 to 1:3200. A possible drug-induced lupus erythematosus was recently reported in a patient receiving a combination of emtricitabine, rilpivirine, and tenofovir disoproxil fumarate.^21^ Rheumatic symptoms improved after cessation of antiretroviral therapy but re-appeared after it was restarted along with increasing anti-dsDNA titers.

Up to 30% of HIV-infected patients present with renal manifestations. The proportion of lupus-like glomerulonephritis is low. Chang at el. studied 4 patients with concurrent SLE and HIV and reviewed 7 literature reported cases. Half of them had perinatal infection and displayed all classes of lupus nephritis, as well as HIV-associated nephropathy (HIVAN).^10^ Lupus-like nephritis arises either isolated or in the context of systemic lupus disease. Zhang et al. found lupus-like syndromes in 10 out of 98 Chinese HIV patients.^7^ Five of them had renal involvement. In a French study, out of 60 HIV infected patients who underwent renal biopsy, 10 were found to have lupus-like glomerulonephritis.^22^ In another series, 14 patients with lupus-like glomerulonephritis were found among 77 renal specimens from HIV patients.^23^ Ten of them developed end-stage renal disease, and most had proteinuria in the range of nephrotic syndrome. Corticosteroids and mofetil are useful in the treatment of lupus nephritis in these patients.^13^

Mostly, renal disease in HIV stems from systemic inflammation and direct cytotoxicity^24^ and presents as rapidly deteriorating renal failure (HIVAN), especially in black African and Afro-Caribbean patients, late in the course of the disease.^25^ Histology reveals collapsing focal segmental glomerulosclerosis (FSGS) and highly active retroviral therapy can result in a full restoration of renal function. As a result, HIVAN is presently declining while other causes of renal disease are increasing, such as therapy-related nephrotoxicity and aging.

Many studies show that HIV patients display a high frequency of autoantibodies compared to the general population. ANA (antinuclear antibodies) are identified in 17–33% of HIV patients,^26–28^ but in lower titles than SLE and rarely with dsDNA positivity.^14,27,29^ On the other hand, there are patients with SLE who have lost dsDNA positivity after a HIV infection.^14,15^ Other commonly presenting antibodies are anticardiolipin antibodies and ANCAs. The autoantibodies seem to correlate with reduced number of CD4 lymphocytes, but not with hypergammaglobulinemia; the latter being an extremely common finding in HIV patients, both untreated and treated.^27,28^

The biggest clinical challenge is to reach a definite diagnosis of lupus syndrome in a HIV patient who already has non-specific auto-antibodies, because the patient may need immunosuppressive therapy. On the other hand, a pseudo- SLE diagnosis in a patient with undiagnosed HIV infection can be fatal, as the viral infection will progress under immunosuppressive treatment.^30^ SLE diagnosis is generally supported by the presence of anti-dsDNA antibodies, the hypocomplementemia or the histologically proven lupus nephritis.

Inversely, investigation for HIV infection should proceed cautiously in SLE patients, due to cross-reactivity and the possibility of false positive anti-HIV antibodies by ELISA.^31^ More specific assays like western blot, immunofluorescence assay and PCR (Polymerase Chain Reaction) should be employed. In patients with recurrent opportunistic infections, suspicion should prompt a HIV investigation, even though the co-existence of these two diseases is very rare.

## SJOGREN-LIKE SYNDROME

In recent years, Sjögren’s syndrome (SS) has become a rare occurrence in HIV patients,^4,9^ but it used to be a classic presentation of the diffuse infiltrative lymphocytosis syndrome (DILS). DILS is a clinicopathologic entity associated with untreated or poorly controlled HIV infection, described first by Solal-Celigny et al. in 1985.^32^ It is now considered a rare syndrome that develops in certain HIV patients who respond to infection by CD8+ lymphocytosis, with CD8 positive T-lymphocytes infiltrating various organs, especially the salivary glands. Sicca symptoms, both xerophthalmia (dry eyes) and xerostomia (dry mouth), with or without parotid gland swelling, are frequently part of DILS, and the whole presentation resembles that of SS.^33^ DILS can also affect extraglandular tissues, in contrast to SS. AIDS diagnosis belongs in the exclusion criteria for primary SS (American College of Rheumatology/European League Against Rheumatism 2016 classification criteria).^34^

In a 2003 Greek study, by Panayiotakopoulos et al., the incidence of SLS dropped from 7.8% in the pre-HAART era to 1.5% post-HAART, which is actually lower than in the general population, meaning that compliance to therapy practically eliminated SLS.^35^ These patients, even though they reported symptoms of xerophthalmia, tested negative in Rose-Bengal staining test, had positive parotid scans, but only 2 out of 17 had histologically proven SLS. Primary therapy for SLS is the HIV infection control. **Table 1** describes the differences between SS and DILS.

**Table 1.** Differences between SS and DILS.

	SS	DILS
Demographic	Middle aged/elderly women	Young men
Sicca symptoms	+	+
Anti-SSA/Ro, anti-SSB/La	+	−
Schirmer’s test, BUT test	+	+
Rose Bengal staining	+	−
Positive parotid gland scanning	+	+
Typical salivary gland biopsy	+	−
Lymphocytic infiltrates	CD4	CD8

## VASCULITIDES

Chronic viral infections have long been associated with vasculitides. The incidence of vasculitis in HIV patients is estimated at less than 1%.^36^ Pathogenesis is associated with direct endothelial cell damage, cell-mediated toxicity, immune complexes or autoantibodies. A wide range of vasculitides affecting large, medium and small vessels have been described in HIV-infected patients, from those triggered by opportunistic infections to non-specific vasculitis.^36^ In many cases viral eradication is beneficial for the autoimmune disease. Iordache et al. found 4 patients with Takayasu arteritis, 3 with Henoch–Schönlein purpura, 2 with periarteritis nodosa, 1 with cutaneous vasculitis and 1 with granulomatosis with polyangeitis among 52 studied subjects.^20^ Seven of them needed immunosuppressive treatment. Zhang et al. reported 20 cases of various vasculitides among 98 consecutive HIV patients between 1999–2006.^7^ Many of them had a hepatitis C virus (HCV) co-infection. Kawasaki-like syndromes have been reported in HIV patients.^37^ A case of immune restoration cerebral vasculitis, not responding to combined immunosuppressive treatment, but eventually responding to the monoclonal anti-CD25 antibody daclizumab has been described.^38^

### Antineutrophil cytoplasmic autoantibody - associated systemic vasculitis

ANCA-associated systemic vasculitis (AASV) is characterized by the necrotizing inflammation of small blood vessels with paucity of immune deposits. Suspicion of AASV should be raised in patients presenting with multiorgan disease such as renal, skin, pulmonary or neurologic manifestations.

ANCAs are frequently found in HIV patients by indirect immunofluorescence (13–42%), but less often by ELISA assays^27,39^ and they are rarely associated with a rheumatic disease.^39,40^ TNF-alpha produced by monocyte-macrophages of HIV-infected patients induces antigens like PR3 and MPO and could explain the development of autoantibodies.

An African patient with MPO-ANCA associated pauci-immune glomerulonephritis responding to steroid and rituximab treatment has been reported.^41^ Churg-Strauss syndrome was described as the initial HIV presentation in a young woman.^42^ In 1 patient with AIDS and AASV the histological examination of lymph nodes revealed EBV positive staining (EBER1), suggesting an etiopathogenic role.^43^ An interesting case of lung disease resembling granulomatosis with polyangiitis (Wegener’s), in a patient which was later found to be HIV positive, provides an insight into the correct evaluation of cANCA positivity.^44^ When the clinical manifestations are severe, as in central nervous system vasculitis, immunosuppressive therapy has to be applied, although it may jeopardize the clinical course of the viral-infected patient.

### Cryoglobulinemia

Cryoglobulins, especially in patients co-infected with HCV have been reported in HIV patients.^39,45–47^ Bonnet et al. detected cryoglobulins in 17% of HIV patients and in 42% of those co-infected with HCV. ^39^ Mixed cryoglobulinemia was reported in 11 patients with both HCV and HIV, who did not respond to HIV antiviral therapy but responded to either anti-HCV treatment or corticosteroids.^45^ Kordossis et al. studied 87 consecutive HIV infected patients for a median of 34 months and showed that mixed cryoglobulinemia was an independent predictor of death, neoplasia or B-cell lymphoproliferative diseases.^48^

### Henoch - Schönlein purpura

The Henoch-Schönlein purpura (HSP) is an IgA-mediated small vessel vasculitis presenting with lower extremities’ palpable purpura, nephritis, arthritis and gastrointestinal disease. Few patients with HIV and HSP have been reported.^7, 49–54^ Most patients were far from antiretroviral therapy (either untreated or interrupted) with low CD4 T-cell counts and had mild or prominent proteinuria with renal impairment along with various combinations of purpuric rash, arthralgia and gastrointestinal symptoms. Most patients responded to corticosteroids and antiretroviral therapy, but in some cases, plasma exchange was needed.^50,52^ In some patients a simultaneous infection with another viral agent like CMV^50^ or HBV^55^ was detected.

A distinct form of IgA nephropathy in HIV patients, that is associated with immune complexes composed of IgA idiotypic antibodies and anti-HIV antibodies, has been described.^55,56^ Patients with HIV often have immune activation with elevated serum IgA and circulating immune complexes which can be related to the pathogenesis of IgA nephropathy; the virus itself may have a direct effect on the vascular endothelium.^49,57^ Most reported cases of HIV-associated IgA vasculitis/nephropathy originated in Asia.

### Adamantiades-Behçet’s disease

Behçet’s disease (BD) is an autoimmune multisystem disease that presents with recurrent oral aphthae, uveitis, skin lesions and painful genital ulcers.^58^ The cause of BD is unknown, but infectious agents either viral (herpes simplex virus and parvovirus B19) or bacterial (streptococcus, helicobacter pylori) and genetic factors (HLA-B51) play a role in its development.^59^ The underlying pathology is blood vessel inflammation with CD4+ T-cell and neutrophilic infiltrates and occasionally thrombosis due to excess thrombin formation.^60^ All sizes of blood vessels and both arterial and venous sides of circulation can be affected. Treatment varies according to organ involvement and severity of symptoms and includes nonsteroidal anti-inflammatory drugs, colchicine, corticosteroids, azathioprine, thalidomide, etc.

Several cases have been reported in relation to HIV infection.^7,61–69^ In general, patients respond to HAART alone^63,64^ or in conjunction with drugs commonly used in the treatment of BD. A patient reported by Siddiqui et al. had recurrent symptoms under HAART and intestinal bleeding that required a colectomy. The patient eventually responded to colchicine, although non-compliance with antiretroviral therapy is mentioned.^68^ In another case, a patient had a multisystem disease that responded partially to HAART and hydroxychrolochine plus colchicine, but later developed IgA nephropathy requiring immunosuppressive therapy.^65^ BD was found in 15% of a cohort of 98 Chinese HIV patients, mostly not under HAART.^7^ HIV infection itself can cause some of the classical BD symptoms like orogenital ulcers, uveitis or folliculitis. The study of the various cases reveals that BD symptoms appear in patients with poor disease control, so the HIV virus must have a direct pathogenic role in the BD vasculitis. Additionally, opportunistic infections may also contribute, as well as intravenous drug use and genetic predisposition.^7,65^ Clinicians are advised to use the diagnostic criteria proposed by the International Study Group for Behçet’s Disease and if possible the detection of HLA-B51 in order to reach a correct diagnosis.^58^

### Cerebral vasculopathy with aneurysm formation

Cerebral vasculopathy with aneurysm formation is a rare condition mostly found in HIV-infected children and occasionally young adults who present with ischemic or hemorrhagic stroke. The pathophysiology is unknown and a direct virus toxicity of either HIV or varicella-zoster virus has been implicated. It spares the small arteries and affects the Willis circle arteries in severe immunocompromised patients. Angiography shows diffuse fusiform aneurysms. Therapeutically, an adequate viral control with anti-retroviral therapy is warranted, whereas corticosteroids have an undefined role.^70^

## ANTIPHOSPHOLIPID SYNDROME

Antiphospholipid syndrome (APS) is an autoimmune disease that presents with thrombotic or obstetric complications and less frequently with cytopenias (mainly thrombocytopenia) in association with non-temporary, high-titer antiphospholipid antibodies (APLAs) and/or lupus anticoagulant activity in the sera of affected patients. In these patients, APLAs display specificity for a neoepitope comprising of a complex between the beta2-GPI, a lipid-binding coagulation inhibitor, and cellular membrane phospholipids, thus having a direct role in thrombosis.^71^ Many viral, bacterial and parasitic infections are associated with temporary development of non-clinically significant anticardiolipin antibodies (aCLs).^72^ These aCLs are not specific for β_2_GPI, but are directed against cellular membrane lipids, so they display no thrombotic potential.^72^

Anticardiolipin antibodies and anti-beta2 GPI antibodies are often detected in HIV-infected patients, but rarely cause symptoms of the APS.^73–76^ Their incidence is up to 50%, correlates with the viral load and the degree of immune system activation and has diminished in the HAART era.^73^ They may be driven by increased cellular lipid oxidation.^77^ A 2009 French review found aCLs in 50% of HIV patients, but only 5.6% with anti-b2GPI, in contrast with a Brazilian study, in which an anti-b2GPI positivity (primarily of the IgA isotype) was found in 33% of the studied patients.^74,75^ Similarly, Petrovas et al. found anti-beta2 GPI in 5% of their study population, all without features of APS.^77^ Lupus anticoagulants (LACs) are also detected in AIDS patients albeit at a lower rate (3% in a 2009 Nigerian study, with no APS manifestation).^78^

In a 2004 review, 17% of patients with infection-associated APS had HIV infection.^72^ Galrão et al. found 44% of HIV patients with at least one type of antiphospholipid antibody, but only 13% with an APS manifestation.^74^ Iordache et al. identified 4 patients with APS among 52 subjects with HIV-associated ADs.^79^ Several case reports are found in the literature with various APS manifestations, such as pulmonary embolism,^80^ deep or superficial vein thrombosis,^81^ arterial thrombosis,^74,82^ cutaneous manifestations.^82,83^ Avascular bone necrosis has been described in a number of patients and seems to be a unique HIV/APS feature.^82,84^ Although HIV infection increases the risk of miscarriage we did not find any case report with APS-associated obstetric complications in infected women. Management of APS depends on the symptoms and consists basically of anticoagulant therapy. In cases of autoimmune cytopenias or catastrophic APS, immunosuppressive therapy has also been incorporated.

## CONCLUSIONS

The increased occurrence of rheumatic disorders in patients infected with HIV has been attributed to various characteristics of the virus itself, as well as the disruption of immune system regulation and the reaction to acute and chronic infection and to highly active retroviral therapy. These mechanisms include direct viral cytotoxicity, molecular mimicry, activation of B lymphocytes with production of autoantibodies especially in case of triggering opportunistic infections, disruption of the normal Th1 and Th2 balance, increased cytotoxic T-cell response and the immune reconstitution inflammatory syndrome.^1,4^ The natural history of the disease has been altered dramatically by the implementation of protease inhibitor-based antiretroviral therapy in 1996. The reduction in disease mortality and morbidity lead to the emergence of a new spectrum of autoimmune disorders. For some of them, HAART is a sufficient management, while in more severe cases immunosuppressive therapy is warranted. Caution is recommended in interpreting clinical and laboratory findings and discriminating actual rheumatological disorders from non-specific manifestations of acute and chronic HIV infection.

## ABBREVIATIONS

AD: Autoimmune diseases
AIDS: Acquired Immune Deficiency Syndrome
ANCA: Antineutrophil cytoplasmic autoantibody
BD: Behçet’s disease
CTD: Connective tissue disorders
HAART: Highly Active Anti-Retroviral Therapy
HIV: Human immunodeficiency virus
HIVAN: HIV-associated nephropathy
HSP: Henoch-Schönlein Purpura
IRIS: Immune reconstitution inflammatory syndrome
SLE: Systemic Lupus Erythematosus
SLS: Sjögren-like Syndrome
SS: Sjögren’s Syndrome
